# Blowing on Embers: Commensal Microbiota and Our Immune System

**DOI:** 10.3389/fimmu.2014.00318

**Published:** 2014-07-28

**Authors:** Darina S. Spasova, Charles D. Surh

**Affiliations:** ^1^Kellog School of Science and Technology Doctoral Program in Chemical and Biological Sciences and the Department of Immunology and Microbial Science, The Scripps Research Institute, La Jolla, CA, USA; ^2^Division of Developmental Immunology, La Jolla Institute for Allergy and Immunology, La Jolla, CA, USA; ^3^Academy of Immunology and Microbiology, Institute of Basic Science, Pohang, South Korea; ^4^Department of Integrative Biosciences and Biotechnology, Pohang University of Science and Technology, Pohang, South Korea

**Keywords:** commensal, microbiota, immune system, T cells, regulation

## Abstract

Vertebrates have co-evolved with microorganisms resulting in a symbiotic relationship, which plays an important role in health and disease. Skin and mucosal surfaces are colonized with a diverse population of commensal microbiota, over 1000 species, outnumbering the host cells by 10-fold. In the past 40 years, studies have built on the idea that commensal microbiota is in constant contact with the host immune system and thus influence immune function. Recent studies, focusing on mutualism in the gut, have shown that commensal microbiota seems to play a critical role in the development and homeostasis of the host immune system. In particular, the gut microbiota appears to direct the organization and maturation of lymphoid tissues and acts both locally and systemically to regulate the recruitment, differentiation, and function of innate and adaptive immune cells. While the pace of research in the area of the mucosal–immune interface has certainly intensified over the last 10 years, we are still in the early days of this field. Illuminating the mechanisms of how gut microbes shape host immunity will enhance our understanding of the causes of immune-mediated pathologies and improve the design of next-generation vaccines. This review discusses the recent advances in this field, focusing on the close relationship between the adaptive immune system and commensal microbiota, a constant and abundant source of foreign antigens.

## Introduction

Microorganisms represent the oldest and most ubiquitous forms of life on our planet. These microbes evolved alongside other organisms, like metazoans, with a portion of them colonizing and establishing life-long relationships with larger species. Such mutualistic relationships have been described in plants (1), insects (2), worms (3, 4), fish (5, 6), birds (6), and mammals (7). Shortly after birth, the mammalian mucosal tissues are exposed to the environment and colonized by viruses, bacteria, protozoa, and fungi, resulting in the formation of complex populations of microbes, collectively called the commensal microbiota. The mammalian commensal microbiota constitutes of over 1000 different species of microbes and outnumbers the host cells by 10-fold (8, 9). The intestinal mucosa harbors the largest amount of microbes in the human body; many are beneficial while some, termed pathobionts, are generally harmless, but can turn pathogenic with an imbalance of the microbial community. The commensal microbiota has long been appreciated for its essential contributions to host development and health. Such benefits include metabolism of indigestible food byproducts, generation of nutrients, defense against pathobionts, promotion of angiogenesis and enteric nerve function, maintenance of intestinal structure, and contribution to the development and regulation of the mammalian immune system (10–15).

For better and for worse, the mammalian immune system is one aspect of host physiology that is heavily influenced by commensal microbiota. Both human and animal model research supports the hypothesis that immune-related disorders like inflammatory bowel disease (IBD) (16), cancer (17), diabetes (18, 19), allergies (20), and even obesity (21, 22) may result from dysbiosis of the commensal microbial communities. To better understand how microbiota contributes to the onset and/or exacerbation of such disorders (23, 24), it is important to understand how signals from intestinal microbiota influence the immune system under normal and diseased conditions. This review discusses the effect of gut microbiota on the mammalian immune system, with a particular focus on T cell differentiation, responses, and homeostasis. First, we discuss how the host senses microbes. Then, we review the ability of dendritic cells (DCs) to induce immunogenic or tolerogenic responses to microbial signals. Finally, we examine the signals that microbes provide and their role in modulating T cell differentiation and function.

## Host Sensing of Microbes

Commensal microbiota may be largely harmless but it is nevertheless crucial to maintain barriers to prevent opportunistic invasions and, occasionally, an immune response is required to keep potential pathobionts in check. In mucosal tissues, such a response is promoted by both epithelial and innate immune cells via ligation of various receptors expressed differentially in these cells. The recognition of microbial particles such as DNA, cell wall components, and metabolites occurs in the context of innate toll-like receptors (TLRs), NOD-like receptors (NLRs), G protein-coupled receptors (GPCRs), other pattern-recognition receptors (C-type lectin receptors, RIG-I-like receptors, and AIM-2-like receptors), and yet to be identified receptors expressed by both hematopoietic and non-hematopoietic lineage cells in mucosal tissues. The following section will focus on the use of TLRs and NLRs by the host to sense microbes.

### Toll-like receptors

Toll-like receptors are pattern-recognition receptors, which bind to evolutionarily conserved molecules expressed by bacteria, viruses, and other microorganisms (25). Some of them (TLRs 2, 3, 4, and 5) are expressed on the cell surface, whereas others (TLRs 3, 7, and 9) are expressed in endosomal compartments of cells (26, 27). TLRs are expressed in high levels on epithelial cells and innate immune cells and can sense various microbial molecules such as double- and single-stranded RNA, LPS, flagellin, CpG (28), ensuring detection of all microbes.

Toll-like receptor ligands have been implicated in the onset and management of various diseases. For instance, TLR signaling has been shown to affect Crohn’s disease and psoriatic arthritis in humans (29, 30). Studies from the non-obese diabetic (NOD) murine model of type-1 diabetes have also revealed that disruption of TLR signaling protects these mice from developing diabetes (31). These data indicate that microbial recognition via TLRs has a significant implication in the onset of autoimmune and inflammatory diseases.

Toll-like receptor ligands have also been suggested to modulate mucosal cell function and tissue maintenance. One mechanism is by inducing the synthesis of antimicrobial peptides and tissue-repair factors by intestinal epithelial cells (IECs) (32). Moreover, LPS-induced TLR signaling has been linked to NFκB activation of IECs, which results in promoting either a tolerogenic or a pro-inflammatory environment depending on the microbial context (33). LPS also acts directly on mucosal DCs, affecting their activation and migration to various anatomical sites where they can induce adaptive responses (33). Similar to IECs, TLR signals can induce either activation or tolerance programs in DCs (discussed below).

### NOD-like receptors

NOD-like receptors contain multiple subfamilies and are expressed by many organisms from plants to mammals. Mammalian NLRs play a crucial role in sensing microbes and inducing various pro-inflammatory programs in response to microbial products (34). NLRs are not only essential in responses to pathogens but they are also necessary for the development of intestinal lymphoid tissues, maintenance of commensal communities, and mounting antigen-specific immune responses (35). This section will discuss only the NLRC and NLRP subfamilies of NLR signaling. For a more comprehensive discussion of NLRs, see the recent review by Chen et al. (36).

The NLRC subfamily contains NOD1, which recognizes peptidoglycan-containing mesodiaminopimelic acid (iE-DAP), and NOD2, which recognizes muramyl dipeptide (MDP) (37). One of the major functions of NOD1/2 is the activation of NFκB signaling. Pathogen induction of NOD2 results in NFκB activation and secretion of pro-inflammatory cytokines, especially IL-1 (34). Mutations in NOD2 are linked to Crohn’s disease; here, the inability to induce NFκB signaling on IECs and immune cells is thought to impair intestinal mucosal barrier function, resulting in pathologies from invasion of the intestine by commensal bacteria (38). Support for this notion was obtained from work in the mouse. Hence, increased intestinal permeability to commensal bacteria was reported in NOD2 deficient mice, indicating that NOD2 signaling is important for maintaining intestinal epithelial integrity and barrier function (39). Similarly, NOD1 also has a role in maintaining intestinal homeostasis but in other related ways. Thus, NOD1 deficient mice possess a decreased number of intestinal lymphoid follicles and lower expression of CCL20 as well as a change in microbiota composition (35). NOD1 and 2 signaling has been implicated in both pro-inflammatory and immunoregulatory roles as it can induce secretion of IFN-γ, IL-12, IL-6 but also of IL-10 in various models of disease, demonstrating thus suggesting that a fine balance of NLRC signaling must be maintained (40–43). It is clear that NOD1 and NOD2 contribute to regulatory and inflammatory responses to microbes but it is the integration of these signals and those from other receptors, such as NLRP, that likely result in determining whether a regulatory or inflammatory response is appropriate.

NLRP and NLRC signaling are closely associated with the immune system as they affect inflammasome function. The inflammasome is a multiprotein complex that is required for the enzymatic processing of pro-forms of IL-1β and IL-18. Multiple NLRs can activate the inflammasome but they all recognize bacterial flagellin or type III secretion system (44). It has been suggested that NLRP and NLRC signaling is more specific than NOD1/2 signaling since it is triggered not only by these two types of bacterial molecules but it also has regulatory molecules in place that are recruited only by certain species of bacteria that merit an inflammatory response (44, 45). A recent model proposes that inflammasome activation to various microbes is mediated commonly by NLRC4, but the specificity comes from differential NLRP recognition of products from specific bacterial species (46). NLRP4, NLRP3, and NLRP1 have been associated with induction of the inflammasome in response to bacterial, fungal, and viral products; mutations in these genes are associated with higher susceptibility to bacterial and viral infections as well as autoimmunity (47–49).

Even though NLRP activation of the inflammasome was believed to be largely in response to infection and severe cellular stress, it was recently demonstrated that NLRP6 plays a crucial role in regulating interactions with commensal microbiota during steady-state conditions. NLRP6 knockout animals were highly susceptible to DSS-induced colitis and had decreased intestinal IL-18 levels as well as altered microbiota composition (50). It is unclear exactly what triggers NLRP6-mediated activation of the inflammasome but it likely occurs in response to a collection of environmental signals. Because NLRP activation can occur in the context of microbiota, stress, or infection, the discrimination between pathogenic and commensal microbes likely involves the integration of multiple signals from the inflammasome, NFκB signaling, and other pathways by innate immune cells.

## Innate Lymphoid Cells

Innate lymphoid cells (ILCs) comprise a recently described population of innate immune cells, which have been shown to regulate immunity, inflammation, and tissue repair in various anatomical sites (51–53). Like B and T cells, they differentiate from a common lymphoid progenitor but, unlike B and T cells, they lack a rearranged antigen-specific receptor (51–53). Nonetheless, ILCs closely resemble CD4 helper T cells and express many of their transcription factors and molecules. Multiple subsets of ILCs exist, and they are classified into three groups – Groups 1, 2, and 3 – according to the expression of distinct transcription factors and effector molecules (51–53). Group 1 ILCs, such as NK cells, are characterized by the expression of the transcription factors T-bet and/or Eomesodermin and the production of the cytokine IFN-γ in response to stimulation with IL-12 and IL-18 (51–53). Group 2 ILCs’ canonical features are the expression of the transcription factors RORα and GATA3 and the expression of IL-5 and IL-13 in response to stimulation with IL-25, IL-33, and TLSP (51–53). Finally, group 3 ILCs, such as lymphoid tissue inducer (LTI) cells, NKp46^+^, and NKp46^−^ cells, are characterized by the expression of RORγt and/or T-bet and/or the aryl hydrocarbon receptor (AHR) and upon stimulation with IL-23 and IL-1β, they produce IL-22 and/or IL-17 and/or IFN-γ (51–53).

Innate lymphoid cells inhabit various tissues where they have been shown to direct inflammation at mucosal surfaces, especially in a response to infection, and confer resistance to pathogens (54). Group 1 ILCs include NK cells and a population of human mucosal ILCs, which lack expression of NK surface markers but produce high levels of IFN-γ in response to IL-18 and IL-12 stimulation (55). They are present in high levels in patients with Crohn’s disease and contribute to increased inflammation in the intestine (56). A new NKp44^+^CD103^+^ population of ILC1s, which produces large amounts of IFN-γ, CCL4, and TNFα, has been recently described in the human intestine (57), and it has been suggested that it is the innate counterpart of tissue-resident memory CD8 T cells. Group 2 ILCs participate in protection against helminth infection in the intestine by producing IL-25 (58–60) as well as in wound healing in the airways via the production of amphiregulin, which induces epithelial cell proliferation (61, 62). Group 3 ILCs have been shown to regulate CD4 T cell responses to commensal bacteria in the intestine of mice as well as to prevent bacterial dispersal to peripheral tissues (63, 64). Recently, it has also been revealed that intestinal macrophages act through ILC3 to induce the production of tolerogenic molecules (IL-10 and retinoic acid) by intestinal DCs or macrophages and thus promote the induction and homeostasis of regulatory T cells in the intestine and the mesenteric lymph nodes (65). Even though ILCs are a novel population of innate immune cells, it has been well demonstrated that they play a key role in mucosal defense from pathogens as well as in maintenance of mucosal tissues.

## Dendritic Cell Function Depends on Location and Environment

Mucosal DCs are in constant contact with commensal microbiota via various pattern-recognition receptors. Because DCs have the unique ability to elicit a robust T cell response (66), they are differentially conditioned based on anatomical location and local antigenic load to be either tolerogenic or pro-inflammatory in response to the same signal. In the lungs, TLR4 signaling on DCs results in antigen-specific CD4 T cell mediated inflammation (67). On the contrary, TLR4 signaling in intestinal DCs is shown to be tolerogenic (68), suggesting that the environmental cues and location determine how a DC integrates such signals. In the context of other TLR ligands, administration of CpG in TLR4 deficient mice reduces their susceptibility to systemic allergy development, arguing for a tolerogenic role of TLR9 signaling (69). In contrast, when CpG is administered to germ-free mice, it causes increased IFN-γ and IL-17 production in the intestine of these mice, conferring protection against intestinal parasites, indicating a more activating role for TLR9 (69). Based on these data, it is widely accepted that the environmental context in which DCs integrate the same signal determines whether an inflammatory immune response occurs.

At mucosal sites, unlike in other lymphoid tissues, DCs perform unique roles and occupy distinct niches (70). In addition to specifying the inflammatory or anti-inflammatory nature of the T cell response, DCs also direct the homing of effector cells. In the mesenteric lymph node, they can promote upregulation of CCR9 and α4β7 on B and T cells (71, 72), thereby facilitating their migration back to the intestine (73, 74). In the intestine, DCs translocate to and reside in the lamina propria (LP), Peyer’s patches, isolated lymphoid follicles, and mesenteric lymph node, where they sample and present luminal and self antigens to T cells (75). In the Peyer’s patches, DCs are divided into three groups: CXCR1^+^ DCs, CCR6^+^ DCs, and CCR7^+^ DCs. Each group has distinct functions and characteristics. CXCR1^+^ DCs are found in the Peyer’s patch in close proximity to M cells, positioned there with the purpose to sample luminal antigens in a TLR-dependent manner (76). CCR6^+^ DCs are migratory and are found in the dome of the Peyer’s patches, from where they readily translocate to the follicle-associated epithelium in response to microbial stimulation. Finally, CCR7^+^ DCs are found in the T cell areas of the Peyer’s patches, where they can induce T cell activation (77, 78) and migration (73, 74), and IgA production by B cells (79, 80) in response to microbial signals (81).

As Peyer’s patches are rare along the intestinal tract, the majority of gut-associated DCs are more frequently found in the LP of the small intestine. LP DCs, often differentiated from circulating precursors (82), express tight junction proteins that allow them to reach their dendrites between IECs and sample the luminal contents directly (83). This phenomenon depends on CX3C chemokines and TLR ligation (84, 85). CX3CR1 deficient mice exhibit impaired luminal sampling by DCs and are thus more susceptible to *Salmonela typhimurium* infection (85). This finding suggests that luminal sampling by DCs may be critical for protective immune responses against intestinal pathogens.

As mentioned above, intestinal DCs are thought to be more tolerogenic than systemic DCs (86, 87). It has been shown that stimulation of intestinal DCs with LPS results in elevated IL-10 secretion, whereas the same stimulation of circulating DCs results in the production of a variety of pro-inflammatory cytokines (88, 89). The mechanisms that condition intestinal DCs to produce IL-10 in response to LPS stimulation, but not systemic DCs, are still largely unknown. It has been suggested that reduced TLR expression (88, 89) and hyporesponsiveness to TLR ligation (68) as well as negative regulation of NFκB pathways via NOD2 signaling play a role in the desensitization of intestinal DCs to microbial antigens (40–43).

Dendritic cells play a key role in promoting T cell differentiation and responses to microbiota in the gut as well as systemically. Some studies have shown that intestinal DCs can transport self and microbial antigens to the mesenteric lymph nodes (90) where they orchestrate T cell activation and differentiation into effector cells. Intestinal DCs are not exclusively tolerogenic and can induce pro-inflammatory T cell responses by secreting IL-12 and IL-23. IL-12 is a master inducer of T helper 1 (Th1) responses (91) and thus plays an important role in Th1-associated autoimmune diseases, such as IBD (92–94). IL-23 has been implicated in inflammatory T helper 17 (Th17) responses (95) in murine models of joint (96) and intestinal inflammation (97–100) as well as psoriasis (101, 102). Conversely, in addition to Th1 and Th17 responses, intestinal DCs can induce T regulatory cells in the context of retinoic acid, TGF-β, and IL-10 in the gut, suggesting that intestinal DCs can serve to direct T cell differentiation so as to induce the appropriate response based on different contexts (75, 86, 87). DCs can sense microbial antigens from commensal and pathogenic microbes and ideally are able to differentiate between the two in order to induce appropriate T cell responses, protecting the host from infection but at the same time creating a tolerogenic environment for the commensal microbiota to thrive.

## Commensal Microbiota Maintains T Cells’ Poised State

Many dramatic effects of commensal microbiota on host T cells have been documented. Following microbial colonization, immune cells are recruited, induced to differentiate and to reside in the gut (13). At steady state, the intestine houses a large number of T cells, which produce IL-17, IL-22, IFN-γ, and IL-10 (69, 103). In the absence of microbiota, there are deficiencies in the production of these cytokines (15, 103), indicating that commensal microbes control their constitutive production in the gut. CD4 T cell numbers have been shown to be decreased in germ-free mice, affecting T helper 1 (Th1) and T helper 17 (Th17) cells, although regulatory T cell (Treg) frequencies remain the same (69, 103). When germ-free mice are conventionalized, a broad Th1, Th17, and Treg expansion occurs and cytokine production is recovered in the gut, indicating that the accumulation of functional CD4 T cells in the gut is microbiota dependent (104). Furthermore, upon infection with a mucosal pathogen, CD4 T cells in the intestine respond to both the pathogen as well as to the commensal microbiota that permeate the intestinal epithelial layer, demonstrating the ability of the adaptive immune system to overcome its tolerance to commensals and its ability to contain pathobionts and opportunistic pathogens (105). Among the CD8 T cell populations in the gut, intraepithelial lymphocytes (IELs) are significantly decreased in germ-free mice, and they are restored upon colonization (106). Because the host needs metabolites and essential nutrients from microbes, yet at the same time it must protect itself from infection, maintaining a balance between pro- and anti-inflammatory T cell populations in the gut is essential.

In addition to affecting the local populations, there is evidence supporting the notion that commensal microbes also influence the generation and function of the host immune system more broadly. Peripheral lymphoid organ structure and function are disrupted in the absence of commensal microbiota (107). Moreover, T cell responses to systemic antigens are also altered in the absence of commensal microbiota. Recent studies have suggested that commensal microbiota keeps the immune system primed and ready to respond during the steady state and conventional mice respond more robustly to infection than germ-free mice (108). This section will discuss the effect of commensal microbiota on T cell responses both at mucosal sites and systemically.

### Th17 cells

IL-17 producing Th17 cells are represented in high numbers in the LP of the small intestine, where they play a role in protection against extracellular pathogens (103). The differentiation of Th17 cells is dependent on their expression of the transcription factor RORγt, and it is driven by signals from TGF-β and IL-21 or IL-6 (109). Additionally, Th17 cells require IL-23 for maturation and survival (110). As the number of Th17 cells is dramatically decreased in the small intestine of germ-free animals, there is a widely accepted notion that commensal bacteria are required to cue Th17 differentiation (103). A TLR-independent mechanism for the promotion of Th17 differentiation in the gut has been suggested, as signals through MyD88 are not necessary for the induction of Th17 cells (103). Furthermore, data from mice mono-associated with segmented filamentous bacteria (SFB) indicate that even a single species of commensal bacteria is enough to direct T cell differentiation toward a Th17 bias (104, 111).

Not only do microbiota-derived signals stimulate the differentiation and accumulation of Th17 cells in the intestinal LP, but they also maintain Th17 cell homeostasis and survival. Studies have indicated that treatment with the antibiotic vancomycin decreases Th17 populations in the small intestine of conventional mice (103, 112), whereas treatment with a complex cocktail of antibiotics, results in a decreased Th17 frequency in the mesenteric lymph node (113). In other mucosal sites, such as the skin, resident commensal microbiota have been shown to induce Th17 and Th1 differentiation to protect the host from pathogens and possible opportunistic microbes (114).

### Regulatory T cells

Maintaining tolerance to resident bacteria is the key to preventing inflammatory diseases in mucosal tissues. Before the importance of tolerance was widely accepted, it was commonly believed that an unknown pathogen was the trigger for IBD. However, successful treatment of the disease with immunosuppressive drugs has lead to the hypothesis that IBD may result from defects in tolerance to otherwise non-pathogenic gut commensals (115–118).

The idea that T cells must be tolerized to commensal microbiota was suggested decades ago, when in an adoptive transfer model, naïve CD4 T cells caused colitis (119) but were held in check by another population of CD4 T cells (118), now known as regulatory T cells (Tregs). For the purposes of this review, Tregs are T cells that express the master regulator transcription factor, Foxp3, and display anti-inflammatory activity, including secretion of TGF-β and/or IL-10 (120). Extrapolation from early studies led to the proposal that regulatory T cells are required to prevent aberrant T cell responses to resident microbes. Scientists were thus surprised when data from the small intestine of germ-free mice emerged to show that commensal bacteria are not necessary for the development of Tregs in that organ (121–123). Furthermore, when germ-free Tregs were assayed, they were able to suppress colitis-like symptoms albeit not as well as conventional Tregs in the adoptive transfer model of the disease (122, 124), indicating that functionally and developmentally, small intestinal Tregs are independent of commensal microbiota. Even though the small intestine houses a large amount of T cells, it is the colon that houses the largest load of microbiota and is the site of colitis observed in these experiments.

Recent studies on the effect of gut microbiota on Treg development and function have elucidated that colonic Tregs are significantly decreased in germ-free mice, indicating that, in the colon, commensal microbiota is the major inducer of colonic Tregs (125, 126). The exact mechanism of how the induction of colonic Tregs occurs remains unknown. It has been suggested that certain species of microbiota, such as Clostridium clusters IV and XIVa, can induce Foxp3^+^ Treg generation in the colon (125). In order to determine whether commensal microbiota is required for the differentiation of naïve peripheral T cells into colonic Tregs, several groups looked at the expression of an Ikaros-family transcription factor, Helios, which is thought to signify thymic origin of Tregs. They showed that in the colons of germ-free mice, most of the Tregs were Helios^hi^, suggesting that these T cells became Tregs in the thymus (so-called thymic or tTreg), whereas their counterparts in conventional mice were mostly Helios^lo^, indicating that these CD4 T cells converted to Tregs in the peripheral tissues (so-called peripheral or pTreg) (125, 127). These results suggested that in conventional hosts, colonic Tregs are differentiated outside of the thymus (presumably in the gut) in response to foreign antigens. Because the use of Helios in the field is still quite controversial (128, 129), other experimental approaches were utilized to confirm the importance of commensal microbiota on colonic Treg differentiation and function. Subsequent work using Neuropilin-1 (Nrp-1) as a marker to detect tTregs confirmed that induction of pTregs in the colon was mediated by commensal microbiota (130). Another study using a fixed TCRβ showed that colonic Tregs utilize different TCRs from systemic circulating Tregs, reaffirming the notion that they recognize distinct gut antigens (127). Additionally, TCRs from colonic Tregs, expressed as transgenes, were unable to induce generation of tTregs, again supporting the notion that colonic Tregs were peripherally induced in response to commensal antigens (127).

As the mechanism is still unknown, multiple models have been offered to explain how microbiota supports an abundance of pTregs in the gut. It has been proposed that preferential expansion of pTregs occurs in the intestine either by microbial components influencing pTregs directly or indirectly through the products of bacterial metabolism, such as short-chain fatty acids (131–133), and from presentation of bacterial peptides (such as PSA) by innate immune cells (134–136) and/or from effector T cells (129, 137, 138). Another hypothesis offers that the foreign antigen load in the gut is so large that, given a limited capacity for antigen presentation, self-peptides are displaced and the increased prevalence of pTregs simply reflects the increased density of their targets (i.e., foreign-peptide/MHC-II) and decreased availability of tTreg targets (i.e., self-peptide/MHC-II) (127). The question of the specificity of small intestinal pTregs still remains open. It is possible that these Tregs are generated in response to self or dietary antigens, as the abundance of these antigens is greater in the small intestine than in the colon.

Fostering a balance between tolerating commensal microbiota and maintaining the ability to mount an immune response to microbial pathogens is crucial for the survival of the host. Despite the seeming bias toward induction of tolerogenic responses in the gut, immune responses still occur readily against pathogens to protect the host from infections. The mechanisms utilized by the host to distinguish between the commensal and pathogenic bacteria are still poorly understood and are under intense investigation.

### Intraepithelial lymphocytes

Because the intestinal mucosa harbors various opportunistic bacteria, the host has evolved the ability to house cytotoxic killer T cells in close proximity to potential sites of pathogen entry, like the intraepithelial layer of the intestine. The lymphocytes that reside there, also known as IELs, are composed of CD8 T cells that are recruited to and remain in that compartment for the duration of the host’s life. There are three types of IELs, each bearing different characteristics and functions: αβ TCR CD8αα, αβ TCR CD8αβ T cells, and γδ TCR T cells. Whereas αβ TCR IELs respond mainly to pathogenic challenge of the epithelial mucosa, γδ TCR IELs participate in would healing and tissue repair by producing pro-inflammatory cytokines and chemokines and recruiting neutrophils, eosinophils, and T cells.

αβ TCR IEL differentiation and maintenance depend on TLR recognition of bacterial signals as MyD88 deficient and germ-free mice exhibit diminished numbers of these cells (139–141). Functionally, in germ-free and antibiotic-treated mice, the cytotoxic activity of αβ TCR IELs is significantly decreased when compared to conventional mice (140), a phenotype that can be rescued by supplying exogenous endotoxin. This suggests that αβ TCR IEL function is both induced and maintained by ubiquitous bacterial components (139, 142).

Similarly, recent data have elucidated the dynamic relationship between commensal bacteria and γδ IELs during mucosal injury. γδ TCR IELs restrict the spread of bacteria to the mesenteric lymph node following intestinal injury as shown by the lack of such a response in γδ TCR IEL-deficient mice (143). γδ TCR IELs exhibit their functions by producing keratinocyte growth factor, which causes epithelial cell proliferation and restoration of barrier functions in the gut (144). Germ-free animals, even though they have a similar numbers of γδ IELs as their conventional counterparts, have significantly decreased ability to promote mucosal injury repair and prevention of invasion by opportunistic pathogens (143). This problem is evidenced by their decreased ability to produce antimicrobiotal peptides, such as RegIIIγ, as well as pro-inflammatory chemokines and cytokines, such as IL-1β, KC, and MIP2α (143), indicating that commensal microbes are required to promote γδ IEL function.

Continued research will reveal the crosstalk between microbiota and these two populations of IELs as they directly relate to intestinal homeostasis. IELs are able to produce both anti- and pro-inflammatory cytokines and promote both strong cytotoxic and tissue-repair responses.

### Systemic T cell responses to infection

Effects of gut microbiota on innate immune responses to systemic viruses and bacteria have been well demonstrated in the literature (108, 145–147). However, the mechanisms whereby microbiota affects non-mucosal T cell responses have been difficult to comprehend as peripheral T cells are not in direct contact with commensal bacteria. Because T cells confer long-term protection and memory against pathogens, understanding the role of microbiota on T cell responses to infection is crucial. Studies from the 1970s indicated that germ-free and conventional animals exhibit similar immune responses to systemic infection by *Salmonela paratyphi* or lymphocytic choriomeningitis virus (LCMV) (146).

A few groups infected germ-free mice with systemic pathogens in the 1990s. Even though they compared mainly antibody production to assess the immune responses in these mice, a speculation can be made that antibody production is directly connected to T cell responses as T cell help is required for class switching and somatic hypermutation. One group, which infected mice with MCMV and subsequently with *Klebsiella pneumoniae*, concluded that germ-free mice were significantly more susceptible to both pathogens and did not clear the infection in various organs at the same rate as SPF counterparts (148). Moreover, they showed increased mortality by bacterial infection (148). Histologically, the spleens and livers of germ-free animals were more severely affected by the infection and recovered more slowly (148). Another group, which infected germ-free mice with *Salmonella typhimurium*, showed that they were more susceptible to systemic infection than conventional controls (149). Furthermore, they noted a decrease in IgG and IgM responses in the germ-free mice. It could be inferred that the inability to clear a viral and intracellular bacterial infections as well as the decreased antibody production is a result of an impaired T cell response in germ-free mice. Indeed, with improved techniques, more recent work has suggested that systemic immune responses are dampened in the absence of commensal microbes.

Germ-free animals were found to have a higher susceptibility to a prolonged and non-limiting *Listeria monocytogenes* infection compared to conventional animals, a phenomenon supposedly due to the inability of germ-free mice to accumulate T cells at inflammation sites (145, 150). Although *Listeria monocytogenes* is naturally an intestinal pathogen, the method utilized in these studies resulted primarily in infection of the host spleen. Likewise, adaptive immune responses to viral infection of non-mucosal sites are also shaped by the presence of commensal microbes. Antibiotic-treated mice generate significantly fewer virus-specific effector CD4 and CD8 T cells when compared to untreated controls during influenza infection (151). Functionally, CD4 and CD8 T cells in antibiotic-treated mice produce lower amounts of pro-inflammatory cytokines, which correlates with increased virus titers in these mice (151). Further investigation by Abt et al. indicated that antibiotic-treated mice more readily succumbed to influenza infection when compared to conventional counterparts (108). T cell responses in antibiotic-treated mice indicated a decrease in CD8 virus-specific T cells, further confirming that commensal microbiota modulates T cell responses to systemic viral infection (108). The authors concluded that the higher susceptibility of antibiotic-treated mice to infection was due to an inability of macrophages to respond to type-1 interferon and limit viral replication. However, they did not expound upon the relationship between the macrophage defect and the CD8 T cell phenotype they observed. It could be inferred that commensal-derived signals provide tonic signaling to innate immune cells, which in turn, influence the ability of these cells to effectively activate naïve T cells and convert them to fully functional effectors. A detailed understanding on the effect of commensal microbiota on systemic T cell responses is yet to be provided.

## Conclusion

Commensal microbiota plays a crucial role in the development, homeostasis, and regulation of the immune system. With the current rise of autoimmune and inflammatory diseases, the importance of inducing and maintaining tolerance to commensal bacteria is increasingly appreciated. As a constant source of foreign antigens, microbiota plays a pivotal role in inducing tolerance to beneficial bacteria as well as in maintaining the immune system poised to defend the host against pathogens. Since the adaptive immune system is often implicated in microbiota-associated inflammatory and autoimmune conditions, understanding its relationship with commensals is crucial. Metaphorically speaking, if the host immune system is a house, the commensal microbes may be likened to the embers in a fireplace. At homeostasis, they remain glowing, providing constant minimal heating for the house. However, when the house so requires, i.e., when an immune response is necessary, they help create a powerful flame.

## Conflict of Interest Statement

The authors declare that the research was conducted in the absence of any commercial or financial relationships that could be construed as a potential conflict of interest.
